# Reduced Motivation in the BACHD Rat Model of Huntington Disease Is Dependent on the Choice of Food Deprivation Strategy

**DOI:** 10.1371/journal.pone.0105662

**Published:** 2014-08-21

**Authors:** Erik Karl Håkan Jansson, Laura Emily Clemens, Olaf Riess, Huu Phuc Nguyen

**Affiliations:** Institute of Medical Genetics and Applied Genomics, University of Tuebingen, Tuebingen, Germany; and Centre for Rare Diseases, University of Tuebingen, Tuebingen, Germany; Emory University, United States of America

## Abstract

Huntington disease (HD) is an inherited neurodegenerative disease characterized by motor, cognitive, psychiatric and metabolic symptoms. Animal models of HD show phenotypes that can be divided into similar categories, with the metabolic phenotype of certain models being characterized by obesity. Although interesting in terms of modeling metabolic symptoms of HD, the obesity phenotype can be problematic as it might confound the results of certain behavioral tests. This concerns the assessment of cognitive function in particular, as tests for such phenotypes are often based on food depriving the animals and having them perform tasks for food rewards. The BACHD rat is a recently established animal model of HD, and in order to ensure that behavioral characterization of these rats is done in a reliable way, a basic understanding of their physiology is needed. Here, we show that BACHD rats are obese and suffer from discrete developmental deficits. When assessing the motivation to lever push for a food reward, BACHD rats were found to be less motivated than wild type rats, although this phenotype was dependent on the food deprivation strategy. Specifically, the phenotype was present when rats of both genotypes were deprived to 85% of their respective free-feeding body weight, but not when deprivation levels were adjusted in order to match the rats' apparent hunger levels. The study emphasizes the importance of considering metabolic abnormalities as a confounding factor when performing behavioral characterization of HD animal models.

## Introduction

Huntington disease (HD) is an autosomal dominantly inherited neurodegenerative disease with a prevalence of 6 per 100,000 in Europe and North America [1]. Development of HD is dependent on a single mutation that results in the extension of the CAG repeat sequence present in the gene for the Huntingtin protein [2]. HD patients display a range of symptoms that can be grouped into motor, psychiatric, cognitive and metabolic symptoms. Symptoms gradually worsen as the disease progresses, and due to the lack of disease modifying treatments HD is invariably fatal.

There are numerous transgenic animal models of HD [3], and as with any disease model, a major focus of working with these is to assess how well their phenotypes mirror symptoms found in HD patients. This is complicated due to the multitude of phenotypes that are often present, and the potential risk of some phenotypes confounding the assessment of others. The metabolic phenotypes are especially interesting in this regard. While HD patients typically lose weight [4], [5], [6], [7], [8], [9], the body weight and body composition phenotypes of transgenic animal models of HD vary [3]. Animals that express the full-length mutant huntingtin gene typically show an increased body weight, due to increased fat mass [10], [11]. Although this is interesting in terms of modeling the metabolic symptoms of HD, an increase in body weight has been suggested to result in reduced performance on the rotarod [12], [13], a common test of motor capacity and limb coordination.

Metabolic phenotypes are also of interest when considering tests of cognitive function, as these are often based on having food deprived animals perform certain tasks to retrieve food rewards [14]. Ideally, animals should be equally hungry and interested in food rewards when performing such tests, as studies where motivational differences are present can give misleading results [15]. Changes in body composition, such as the ones seen in HD models, are likely to either be caused by or lead to a change in *ad libitum* food consumption. Unless careful adjustments are made, such phenotypes might persist even after food deprivation. One proposed method to avoid this when working with HD models is to adjust food deprivation levels until animals show similar consumption rates in tests where they are given brief access to food [16], [17]. Similar tests are occasionally used to assess hunger and food interest, [18], [19], [20], [21] although in HD research one should also consider that a slowed consumption rate could be caused by motor impairments. Thus, detailed knowledge about body composition and feeding behavior of an animal model, both when deprived and *ad libitum* fed, is important for planning and interpreting a variety of behavioral tests.

The BACHD rat is a recently established animal model for HD. These rats carry a large construct containing the full-length gene for human mutant Huntingtin, with its endogenous regulatory sequences [22]. Previous studies have shown that BACHD rats have motor impairments and neuropathological phenotypes reminiscent of symptoms seen among HD patients [22]. In addition, BACHD rats appear to be impaired in some cognitive tests [23]. Previous studies have indicated that BACHD rats eat less than WT rats [22], although the setup used for that particular study demanded social isolation, and its validity for assessing natural behavior has been questioned [24]. Further, although it has been pointed out that BACHD rats appear obese [22], there has not been any study on their body composition. Therefore, we performed a longitudinal study where food intake was measured in a social homecage setup, and body composition was assessed through detailed dissections. As further behavioral characterization of the BACHD rats will be dependent on tests that require food deprivation, we also sought to evaluate an optimal food deprivation strategy for BACHD rats. For this, consumption rate of reward pellets and regular food, as well as performance in a progressive ratio test with prefeedings was assessed at different levels of food deprivation.

## Materials and Methods

### Animals

A total of 168 male rats were used for the study. These were acquired from three separate in-house breeding events, with heterozygous BACHD males from the TG5 line [22] paired with WT females. All animals were on Sprague Dawley background. Animals were genotyped according to previously published protocols [22] and housed in type IV cages (38×55 cm), with high lids (24.5 cm from cage floor), and free access to water. Food availability and social conditions differed between the experimental groups. Rats used for *ad libitum* food intake and body composition measurements were housed in genotype-matched pairs, and had free access to food (SNIFF V1534-000 standard chow) during the entire length of their respective test. Importantly, food was provided on the cage floor and not on the cage top. Body weight was measured weekly to assess general health, and cages were changed twice per week. Rats used for hunger assessment and PR tests were housed in genotype-matched groups of three rats per cage. They had free access to food from the cage top until the age of ten weeks. At that point, the rats were food deprived as described below. Body weight was measured daily in order to assess food deprivation levels, and cages were changed weekly. The animal facility kept 21–23°C, 55–10% humidity, and was set to a partially inverted light/dark cycle with lights on/off at 02:00/14:00 during summer, and 01:00/13:00 during winter.

**Figure 1:**
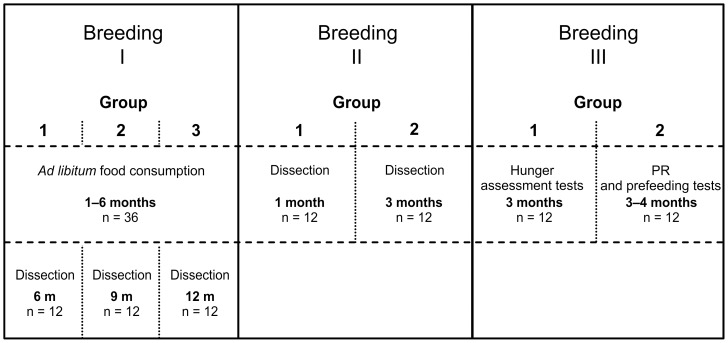
Overview of study groups. A total of seven groups of rats were used in the current study. These were derived from different breeding events and used in different tests, as shown in the figure. The “n” indicates the number of animals used from each genotype. Note that a total of two animals were excluded during analysis, as explained in detail under “Statistical analysis”.

The seven groups of animals were used in different tests, as described below. An overview of the animal groups, and the tests, is shown in Figure 1. All experiments were approved by the local ethics committee (Regierungspraesidium Tuebingen) and carried out in accordance with the German Animal Welfare Act and the guidelines of the Federation of European Laboratory Animal Science Associations, based on European Union legislation (Directive 2010/63/EU).

### *Ad libitum* food consumption in a social homecage environment

*Ad libitum* food consumption was measured using a total of 72 rats, acquired from one breeding event. At the age of five weeks, all rats were arranged into genotype-matched pairs, and housed as described above. This gave a total of 36 cages, 18 cages per genotype. Cages with WT and BACHD rats were evenly distributed over two racks, which were placed next to each other in the same housing room. Food and water intake was assessed twice weekly, when cages were changed. Cages were changed on Mondays and Thursdays during the last two hours of the light phase. At each cage-changing event, a known amount of food was placed inside each new cage, and the fresh water bottles were weighed. The weights of the old water bottles as well as the weight of the food left in each old cage were then measured to assess the amount of food and water consumed since the last cage change. The food was manually collected from the bedding of the old cages. After removing large food pieces, the bedding was sifted in a homemade sieve with a 1 mm mesh in order to collect small food pieces generated by food grinding. The animals' food and water consumption was followed in this way until the age of 26 weeks. Sifting of bedding materials started when animals were 15 weeks old.

### Dissection for body composition assessment

A detailed dissection was performed in order to study the body composition of BACHD rats. Five different rat groups were sacrificed at 1, 3, 6, 9, and 12 months of age respectively, with each group being composed of 12 WT and 12 BACHD rats. The rat groups used for dissection at 6, 9, and 12 months of age were the same rats that were followed during the *ad libitum* food consumption test. The rat groups used for dissection at 1 and 3 months of age were acquired from a separate breeding. Housing conditions were identical for all animals, and according to the description above. Aside from the weekly food and water consumption assessment made during the *ad libitum* food intake test, food and water consumption were measured monthly as animals aged. When rats reached an age of interest, a dissection group was arranged based on the animals' food consumption, water intake, and body weights, so that the dissected group well represented the full group.

Rats were sacrificed in a carbon dioxide chamber two to four hours before dark-phase onset. Blood samples were collected after sacrifice, through retro-orbital bleeding. Body lengths and body weights were measured on the intact animals, with body length measured from nose tip to tail tip. Additional measurements of head, trunk, and tail lengths were measured from nose tip to back of the head, back of the head to anus, and anus to tail tip, respectively. After these external measurements, skin and subcutaneous adipose tissue deposits were removed and weighed. Then, internal organs and adipose deposits located in the abdomen and chest cavities were removed and weighed. The remaining carcass was weighed before removal of the brain. By later subtracting the brain weight, a measurement of bone and muscle weight (denoted bone/muscle) was acquired for each rat. Dissection of a given age group was carried out during four to six days, with rats of both genotypes being assessed on each day.

### Hunger assessment tests

Two tests were used to assess hunger levels in WT and BACHD rats at three different food deprivation levels. A group of 24 animals with equal numbers of WT and BACHD rats was used for both tests. This group was acquired from a breeding separate from the ones used for the *ad libitum* food consumption and body composition measurements. As mentioned above, food deprivation started when the rats were ten weeks old. Body weights were compared to control data from age- and genotype-matched free-feeding animals, on a weekly basis, in order to acquire measurements of food deprivation levels (relative body weight). It should be noted that the control data was not gathered in the current study, but in previous tests. Rats were given small daily amounts of food inside their social homecages, approximately four hours after dark phase onset, to maintain food deprivation. During the first week of food deprivation, animals were habituated to the reward pellets (Bio-Serv, Dustless Precision Pellets® F0021, purchased through Bilaney Consultants, Duesseldorf, Germany) by daily giving each cage a spoon-full of reward pellets together with the daily amount of food. Behavior assessment started one hour after dark phase onset, and was performed in the animals' housing room, using soft red light. Rats were 13 weeks old when behavioral assessment started.

Rats were assessed in both tests on each given testing occasion. The first test assessed the rats' interest in consuming 100 reward pellets. The test used a glass cage (28.5×29×29.5 cm) with mirrors, which allowed a good view of the feeding animals. At the start of each trial, a rat was placed inside the cage, and was allowed two explore it freely during two minutes. Afterwards, a glass Petri dish containing 100 reward pellets was placed inside the cage, in one of the corners that faced the experimenter. The rats were then given a total of five minutes to consume the reward pellets, while the experimenter scored their behavior. The experimenter used two timers to separately record the total time taken to consume the reward pellets, and the time each rat actually spent eating. Thus, one timer was started when the rat first discovered the pellets, and stopped either when all pellets were consumed or when five minutes had passed. The second timer was also started when the rat first discovered the pellets, but was stopped whenever the rat stopped eating, and explored the test arena. Roughly three hours were needed to assess all 24 rats. The test schedule was arranged so that entire cages of BACHD and WT rats were assessed in an alternating manner. Thus, three rats of a given genotype were assessed in sequence, followed by three rats of the other genotype. The experimenter was blinded to the animals' genotypes.

The second test assessed the rats' interest in regular food. In this test, rats were given free access to a large amount of food in their homecages. Food was made available to the rats when four hours remained of the dark phase. Identical amounts of food were placed in the cage tops, with one-minute spacing between cages, alternating between BACHD and WT cages. The remaining food was then measured each half hour, until the end of the dark phase. A final measurement was made at the end of the subsequent light phase. At each measurement, the cages were briefly inspected for larger pieces of food, as they occasionally dropped between the bars of the cage lids.

The rats were assessed in these two tests on three separate occasions. On the first, both WT and BACHD rats were deprived to 85% of their respective free-feeding body weights. In an attempt to reverse the phenotypes that were found, the food deprivation levels were then adjusted so WT and BACHD rats were at 95 and 80% of their respective free-feeding body weights. On the final trial, the previous deprivation levels were switched, so that WT and BACHD rats were at 80 and 95% of their respective free-feeding body weights. Each test occasion was separated by a week of food deprivation, to allow gradual adjustment of deprivation levels.

### Progressive ratio test

A progressive ratio (PR) test was run to assess the rats' motivation to work for a food reward at two different food deprivation settings. A group of 24 animals with equal numbers of WT and BACHD rats was used for the test. This group was acquired from the same breeding as the group used for the hunger tests described above. Food deprivation was initiated and maintained as described above. Behavioral assessment started 30 minutes after dark phase onset, in a room separate from the animals' housing room, using soft red light. Rats were 11 weeks old when behavioral assessment started.

A bank of six operant conditioning chambers (Coulbourn Instruments, H10-11R-TC with H10-24 isolation boxes, purchased through Bilaney Consultants, Duesseldorf, Germany) was used to run the test. Each chamber was equipped with two retractable levers, placed 6 cm above the chamber floor, protruding 2 cm from the wall. The levers were placed on either side of a central pellet receptacle trough, which was placed 2 cm above the chamber floor. The pellet receptacle trough contained a yellow light, which was used to signal the delivery of a reward pellet in all protocols described below. The chambers also contained a red house light, on the wall opposite from the levers and pellet receptacle trough, which shined during the full duration of the training sessions. A water bottle was also available on this wall, to ensure *ad libitum* access to water during testing. All protocols were designed and run with Graphic State 4.1.04. Rats were given single daily sessions, meaning that a total of four daily runs with all six operant chambers were needed to assess the whole group. Each run assessed three WT and three BACHD rats in a determined order, so that a given rat was trained on the same time of day through the entire test. Each rat was assigned to a specific operant chamber, although this was arranged so that each operant chamber was used to assess equal numbers of WT and BACHD rats. Rats received their daily regimen of regular food four hours after the completion of the last run of the day.

During initial training, rats of both genotypes were deprived to 85% of their respective free-feeding body weights. Afterwards, all rats received two habituation sessions in the conditioning chambers. During these, both levers were retracted and a single reward pellet was delivered to the pellet trough at 10, 15, 20, 25, or 30-second intervals. The pellet delivery interval varied in a pseudo-randomized fashion so that each set of five deliveries used each given interval once. Pellet retrieval, or failure to retrieve the pellet within five seconds after delivery, lead to the start of the next pellet delivery interval. After the habituation sessions, rats were trained to lever push for a pellet reward. During these sessions, both levers were extended into the chamber, but only one was reinforced. Rats were either trained to push the right or the left lever, with the reinforced lever position being counter-balanced within the genotype groups. During training, the experimenter would reward rats for approaching, sniffing and touching the reinforced lever, until rats started to reliably push the lever on their own. During this, each lever push was rewarded with one pellet. Training continued until rats completed 100 lever pushes within a 30-minute session, without any help from the experimenter. The rats were then trained on an FR3 protocol, where they had to push the reinforced lever three times before being rewarded with a pellet. When a rat completed 100 ratios within a 30-minute session, it progressed to an FR5 protocol. Rats now had to push the reinforced lever five times before being rewarded with a pellet. Training on the FR5 protocol continued until rats completed 100 ratios within a 30-minute session, on three consecutive sessions. Afterwards, rats were trained on a PR protocol adapted from [16]. In the current protocol, the ten first ratios were of FR5 type. Afterwards, the required number of lever pushes increased after each completed ratio. During this progression, the required number of lever pushes increased in an arithmetic fashion within each block of ten ratios, but also changed between the blocks, to give an overall exponential progression. Thus, during the first, second and third block of ten ratios, the ratio requirement increased with one, three and five pushes per completed ratio, respectively. The PR sessions lasted 80 minutes. The main behavioral parameter of interest was a set of break points, defined as the first ratio where a rat made no responses on the reinforced lever during 10, 25, 50, 100, 300 or 600 seconds. Rats were trained until both genotype groups reached a stable performance, which in this case required 18 sessions. Performance during the six last sessions was defined as baseline performance.

Once stable PR performance had been reached, the rats were challenged in a set of four prefeeding tests. During these tests, the rats were fed specific amounts of reward pellets or regular food, just prior to their daily PR session. Rats were prefed by placing them in individual cages that contained the specified amount of food. Each prefeeding condition was assessed once, in the following order: 100 reward pellets, 250 reward pellets, 4.5 g of regular food, 11.25 g of regular food. Each prefeeding test was separated by two regular PR sessions to ensure that rats returned to their baseline performance.

After completion of the first round of prefeeding tests, the food deprivation level of WT rats was adjusted until they consumed food at the same rate as BACHD rats. Consumption rate was assessed daily by measuring the amount of food consumed during 15 minutes of free access to regular food, placed in the cage tops of the rats' homecages. The rats were still given daily PR sessions during food deprivation adjustments. The food consumption tests were run four hours after completion of the last PR run, i.e. at the time when the rats were usually given their daily food ration. When WT rats had reached a consumption rate equal to that of BACHD rats, six additional PR sessions were run to establish a new baseline. The prefeeding tests were then repeated in the same manner as described above. Rats were 20 weeks old at the end of the test.

### Statistical analyses

All statistical analyses were conducted using GraphPad Prism v.6.01 (GraphPad Software, San Diego California USA, http://www.graphpad.com).

Food consumption in the *ad libitum* food consumption test was analyzed both in terms of the absolute amount of food consumed and the amount of food consumed relative to the animals' body weight. The main analysis of food consumption was based on the weight of large food pieces, as the food debris gathered through sifting of the bedding material also contained hair and bedding pieces. A separate analysis where food consumption was corrected for the amount of food debris was still performed. For this, the mean amount of food debris was calculated for each cage, based on their longitudinal data. This was then added to the weight of the large food pieces measured at each cage changing. For the relative food consumption, rats in a given cage were assumed to eat equal amounts of food. The approximate amount of food consumed by one of the rats was subsequently related to the mean body weight of the two rats. Two-way repeated measures ANVOAs were used to analyze body weight as well as absolute and relative food consumption. Age was used as within-subject factor, and genotype as between-subject factor.

For data gathered in the dissection study, body weight, absolute weight of adipose and bone/muscle tissues, as well as bone/muscle weight relative to body length were analyzed using regular two-way ANOVAs. The factors of interest were still age and genotype. The weights of adipose tissue, bone/muscle tissue and internal organs relative to body weight were analyzed in individual t-tests, or Mann-Whitney tests, between genotypes, within each age group. As the observed phenotypes did not vary between different adipose tissue deposits, only the combined weight of all deposits will be addressed here. One BACHD rat meant for the dissection of six months old animals died before the dissection, making that particular age group 12 WT and 11 BACHD rats.

Results from the two hunger tests were analyzed both within and between each testing occasion. For each test occasion of the reward pellet consumption test, the time needed to consume the pellets was analyzed with t-tests to compare the two genotypes. The time spent exploring the test arena was only analyzed on the first test occasion, using t-test, as rats showed essentially no interest in exploring the arena on later trials. One BACHD rat was excluded from the analysis of the last trial, as he failed to consume all reward pellets within the maximum trial time. The amount of food consumed during the food consumption test was on each test occasion analyzed with two-way repeated measures ANOVA, using time as within-subject factor, and genotype as between-subject factor. To better understand the effect of repeated testing and food deprivation levels, the time needed to consume 100 reward pellets, and the amount of food consumed during the first 30 minutes of the food consumption test were analyzed in additional detail. Thus, data from all three test-occasions were analyzed in two-way repeated measures ANOVAs, using genotype as between-subject factor, and either session number or food deprivation level as within-subject factor. Analysis of baseline performance during the PR test was also made with repeated measures two-way ANOVAs, with break point as within-subject factor, and genotype as between-subject factor. Drops in motivation during prefeeding sessions were analyzed for the 600-seconds break point, as a percentage of the ratio reached during the two preceding PR sessions. Once again, repeated two-way ANOVAs were used to analyze the results, using prefeeding condition as within-subject factor, and genotype as between-subject factor. Separate analyses were performed for prefeeding with reward pellets, and regular food. Bonferroni *post-hoc* test was used to follow up any significant effects of genotype, or interaction effects found in the two-way ANOVAs. Alpha for all analyses was set to 0.05.

## Results

### *Ad libitum* food consumption

**Figure 2:**
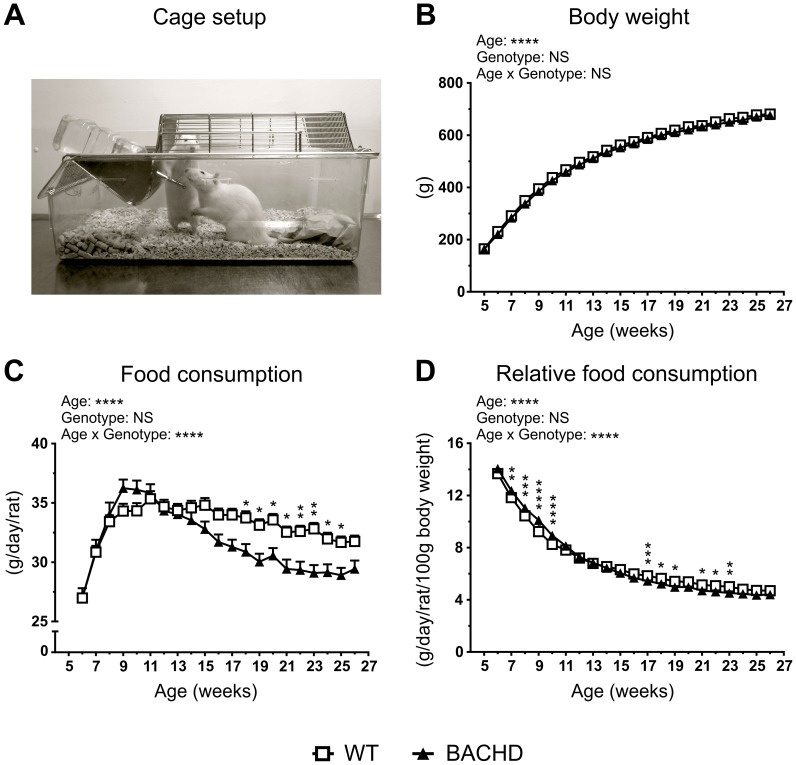
Body weight and food consumption. (**A**) Housing conditions during the *ad libitum* food consumption test. (**B**) Body weight of rats plotted against their age. (**C**) Approximate daily food consumption per rat (calculated from weekly food consumption per cage), plotted against the age of the animals. (**D**) Relative daily food consumption per rat (calculated from weekly food consumption and average body weight per cage), plotted against the age of the animals. The graphs show group mean plus standard error of the mean. Two-way ANOVA results are displayed above each graph, and significant results from *post-hoc* analysis are displayed for individual data points. Genotype differences are indicated by (p<0.05) *, (p<0.01) **, (p<0.001) *** and (p<0.0001) ****.

To assess BACHD rats' growth and food consumption in a low-stress and social environment, we housed genotype-matched rats in pairs (Figure 2A), and measured their weekly body weight and food consumption. Rats of both genotypes grew steadily during the test, as indicated by the significant effect of age on body weight (p<0.0001, F_(21,1449)_  = 2766) (Figure 2B). BACHD and WT rats grew at a similar rate, and showed similar body weights through the entire test, with no significant genotype effect or age x genotype interaction. The rats' food consumption also changed with age (p<0.0001, F_(20,680)_  = 110.5) (Figure 2C). In general, food consumption increased gradually until the age of nine weeks, and then slowly dropped. Importantly, WT and BACHD rats consumed equal amounts of food between six and eight weeks of age, but there were a number of differences seen at older ages. At nine and ten weeks of age, BACHD rats appeared to consume more food that WT rats, although this did not reach statistical significance. Directly following this, food consumption dropped steadily among BACHD rats, while WT rats remained arguably stable until the age of 16 weeks. Due to this, BACHD rats eventually ate less than WT rats, as indicated by the significant results from the *post-hoc* analysis at 17 weeks of age and onwards (p<0.05–0.01). The difference in how food consumption changed with age among BACHD and WT rats was also evident in a significant age x genotype interaction (p<0.0001, F_(20,680)_  = 19.06). Relating food consumption to the rats' body weight gave largely the same results, with a significant age effect (p<0.0001, F_(60,680)_  = 1930) and age x genotype interaction (p<0.0001, F_(20,680)_  = 12.99) (Figure 2D). However, this analysis made the increased food intake among young BACHD rats more apparent, with the *post-hoc* test indicating significant differences between BACHD and WT at seven to ten weeks of age (p<0.01–0.0001). In contrast, the decreased food consumption among old BACHD rats was less apparent, with the *post-hoc* test only indicating a few significant data points at 18 to 21 weeks of age (p<0.05–0.01). It should be noted that BACHD rats produced less food debris compared to WT rats (Figure S1A and B). Correcting for this did not dramatically affect the food consumption phenotype, although the genotype differences became less apparent (Figure S1C). Finally, BACHD rats consumed dramatically less water compared to WT rats (Figure S1D).

### Body composition of BACHD rats

**Figure 3:**
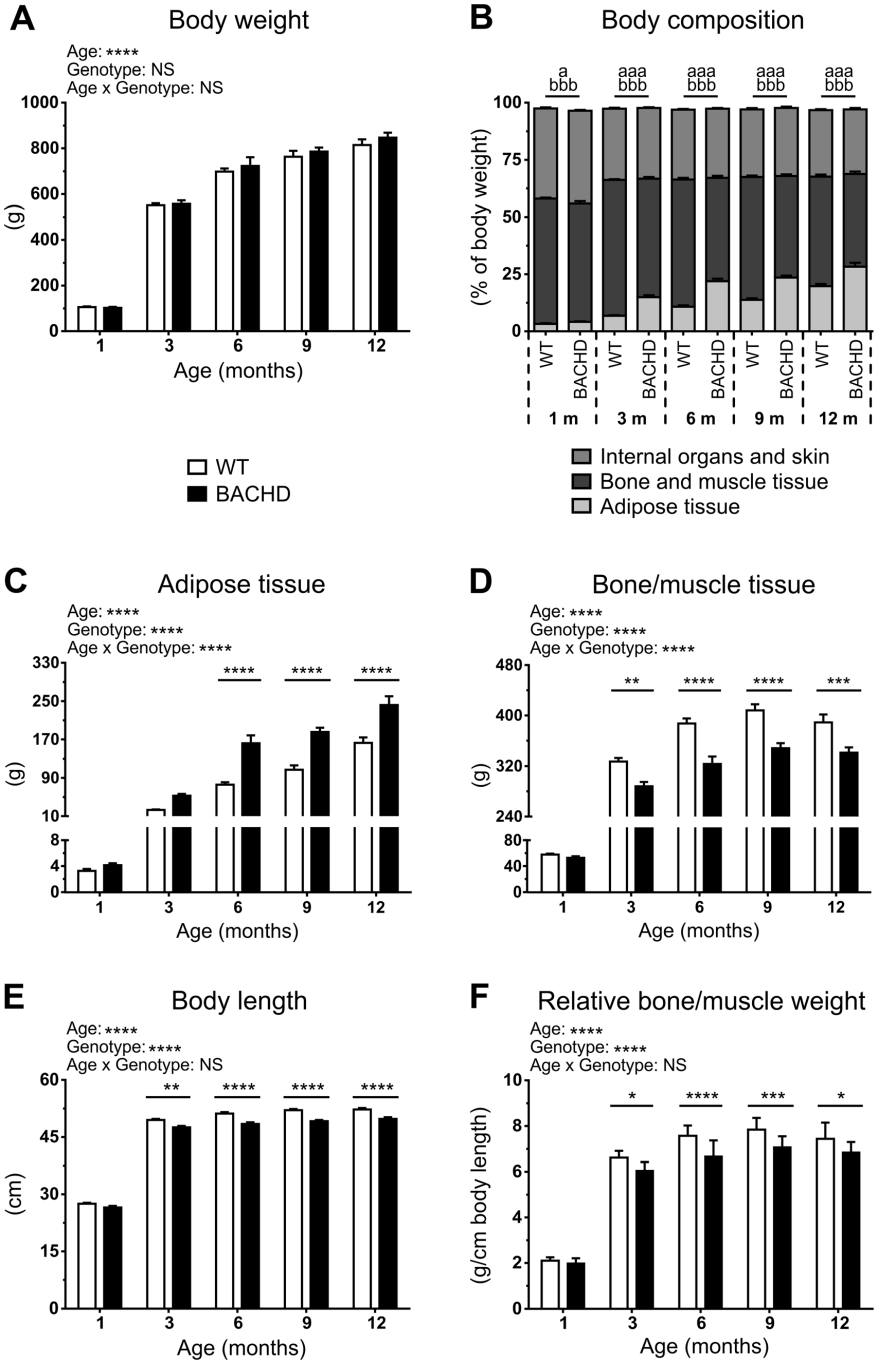
Body composition assessed through dissection. (**A–F**) Data from the dissection groups as stated in the graph titles. The graphs show group mean plus standard error of the mean. Two-way ANOVA results are displayed above each graph, and significant results from *post-hoc* analysis are displayed inside each graph. Significant genotype differences are indicated by (p<0.05) *, (p<0.01) **, (p<0.001) *** and (p<0.0001) ****. For (**B**), ANOVA was not performed, and the indicated differences concern single comparisons between WT and BACHD rats within the age groups. Significant differences are indicated with “a” and “b” for differences in the relative amount of adipose and bone/muscle tissue respectively, written according to the same grading as above.

In order to assess BACHD rats' body composition, we dissected BACHD and WT rats at five different ages. As expected, older rats weighed more, leading to a significant age effect on body weight (p<0.0001, F_(4,109)_  = 444.1) (Figure 3A). In line with previous data, there were no differences in body weight between the genotypes in any age group, and also no significant difference in apparent growth. The body composition of BACHD rats was however different from that of WT rats. BACHD rats had significantly lower percentage of bone and muscle (p<0.001, all ages), and higher percentage of adipose tissue (p<0.05–0.001) in all age groups (Figure 3B). These differences were also apparent when analyzing the absolute weights of the respective tissues. Both WT and BACHD rats gained adipose tissue with age, as indicated by a significant age effect on the weight of total adipose tissue (p<0.0001, F_(4,109)_  = 142) (Figure 3C). However, BACHD rats carried an excess amount of adipose tissue, as indicated by both a significant genotype effect (p<0.0001, F_(1,109)_  = 81.25), and significant results from the *post-hoc* analysis of all groups, except the one-month old rats (p<0.05–0.0001). There was also a significant age x genotype interaction (p<0.0001, F_(4,109)_  = 7.686) that was dependent on data from the one and three months old groups. The bone/muscle weight also increased with age for both genotypes (p<0.0001, F_(4,109)_  = 555.4) (Figure 3D). However, BACHD rats were found to have significantly less bone/muscle tissue compared to WT rats in all but the one-month old age groups. This was indicated both by a significant genotype effect (p<0.0001, F_(1,109)_  = 70.69), and significant results from the *post-hoc* analysis (p<0.01–0.0001). A significant age x genotype interaction (p<0.001, F_(4,109)_  = 4.18) also indicated that there was a difference in the rats' growth. Importantly, this effect was dependent on the data of the one–month old group.

The rats' body length also increased with age for both genotypes (p<0.0001, F_(4,109)_  = 1517), although a significant genotype effect (p<0.0001, F_(1,109)_  = 86.46) and *post-hoc* tests (p<0.01–0.0001) revealed that BACHD rats were smaller than WT (Figure 3E). This was apparent in all age groups except the one-month old animals. It should, however, be noted that one-month old BACHD rats were shorter than WT rats when analyzing litter-matched groups (data not shown). The reduced body length among BACHD rats was mainly due to them having shorter tails and heads compared to WT rats (Figure S2).

BACHD rats also showed a lower amount of bone/muscle tissues in relation to their body length (Figure 3F). Rats of both genotypes gained relative amounts of bone and muscle with age (p<0.0001, F_(4,109)_  = 570.6). However, BACHD rats had lower relative amounts of bone and muscle from three months of age, as evident from a significant genotype effect (p<0.0001, F_(1,109)_  = 47.32) and *post-hoc* analysis (p<0.05–0.0001).

### Assessment of hunger during food deprivation of BACHD rats

**Figure 4:**
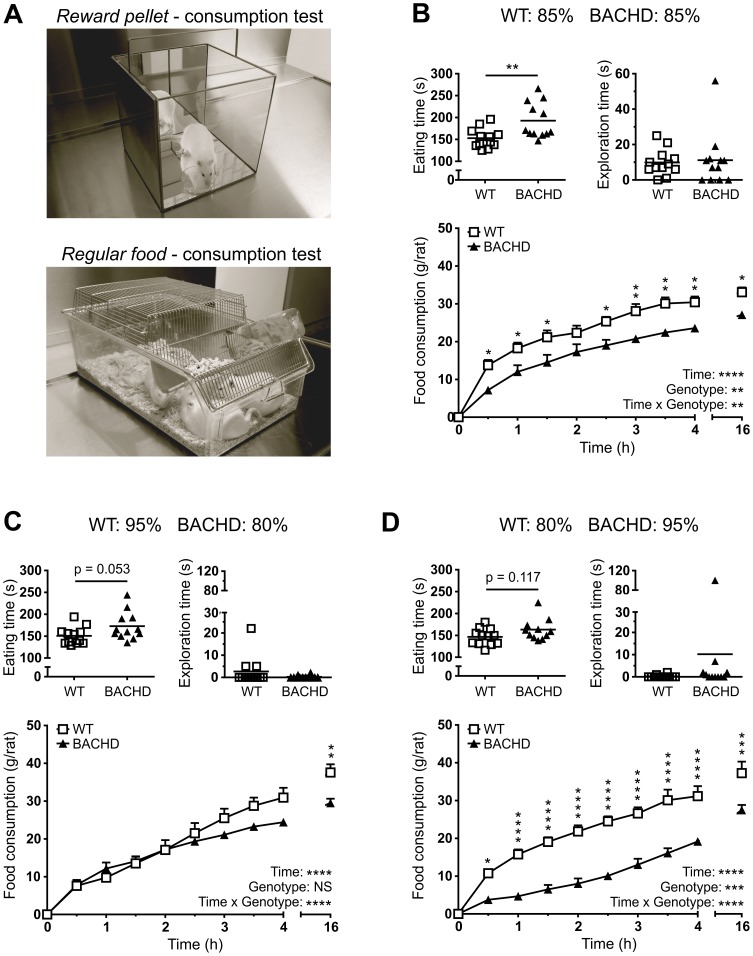
Hunger and food interest assessment. Setups (**A**) and performance in the two consumption tests during the first (**B**), second (**C**) and third test session (**D**), with the different food deprivation levels stated in the title of each figure panel. The time needed to eat 100 reward pellets and the time spent exploring in the reward pellet consumption setup, are displayed in the top left and right graphs of each panel, respectively. The bottom graph of each panel shows the cumulative food consumed per rat during the regular food consumption test. Scatter plots for reward pellet consumption test results indicate individual values and group mean. Line graphs for regular food consumption indicate group mean plus standard error of the mean. Statistical test results are given inside the graphs. For the regular food consumption test, two-way ANOVA results are displayed in the bottom right corner, and results from *post-hoc* analysis are shown for individual data points. Significant genotype differences are indicated by (p<0.05) *, (p<0.01) **, (p<0.001) *** and (p<0.0001) ****.

Two tests based on voluntary consumption of reward pellets and regular food, were run to assess BACHD rats' hunger level at different levels of food deprivation (Figure 4A). When both WT and BACHD rats were deprived to 85% of their respective free-feeding body weights, BACHD rats were found to consume both reward pellets and regular food at a slower rate than WT rats (Figure 4B). In the pellet consumption test, BACHD rats needed longer time to eat the reward pellets (p<0.01), but did not spend more time exploring the arena, compared to WT rats. The slower feeding speed led to a significant increase in trial time for BACHD rats (data not shown). In the food consumption test, BACHD rats were found to have eaten less than WT rats at almost all investigated intervals, as evident from the significant genotype effect (p<0.01, F_(1,6)_  = 14.62), and the significant results from the *post-hoc* analysis (p<0.05–0.01). It should be noted that a difference in actual consumption rate was only seen during the first 30 minutes, resulting in an initial difference in the amount of food consumed, which then persisted through the remaining part of the test. This difference in behavior gave a significant time x genotype interaction (p<0.01, F_(9,54)_  = 2.840) in the amount of food consumed by the rats.

In an attempt to reverse the phenotypes described above, the food deprivation levels were adjusted so that BACHD and WT rats were at 80 and 95% of their respective free-feeding body weights (Figure 4C). In the pellet consumption tests, BACHD rats now needed a similar amount of time to consume the reward pellets, although there was a borderline significant trend towards BACHD rats needing more time (p = 0.0535). With the exception of one WT rat, all rats spent the entire trial eating, and showed minimal interest in exploring the test arena. In the food consumption tests, BACHD and WT rats consumed food at the same rate during the first 150 minutes. During the remaining part of the test, WT rats ate more, eventually leading to a significant difference in the total amount of food consumed during the test (p<0.01). The behavioral differences led to a significant time x genotype interaction effect (p<0.0001, F_(9,54)_  = 8.642).

In a final test, the food deprivation levels were adjusted so that BACHD and WT rats were at 95 and 80% of their respective free-feeding body weights (Figure 4D). At this point, BACHD rats consumed the reward pellets at the same rate as WT rats, as the aforementioned trend was no longer present. With the exception of two BACHD rats, all rats spent the entire trial eating, and showed minimal interest in exploring the test arena. One BACHD rat did not consume all reward pellets within five minutes. In the food consumption test, BACHD rats were once again found to have consumed less food than WT at all investigated intervals, resulting in a significant genotype effect (p<0.001, F_(1,6)_  = 42.52), and significant results from the *post-hoc* analysis (p<0.05–0.0001). BACHD rats ate at a slower rate during the first hour. The consumption rate gradually declined among WT rats, while it gradually increased among BACHD rats, ending up at similar levels after 150 minutes. This difference in behavior gave a significant time x genotype interaction (p<0.0001, F_(9,54)_  = 8.47) in the amount of food consumed by the rats.

**Figure 5:**
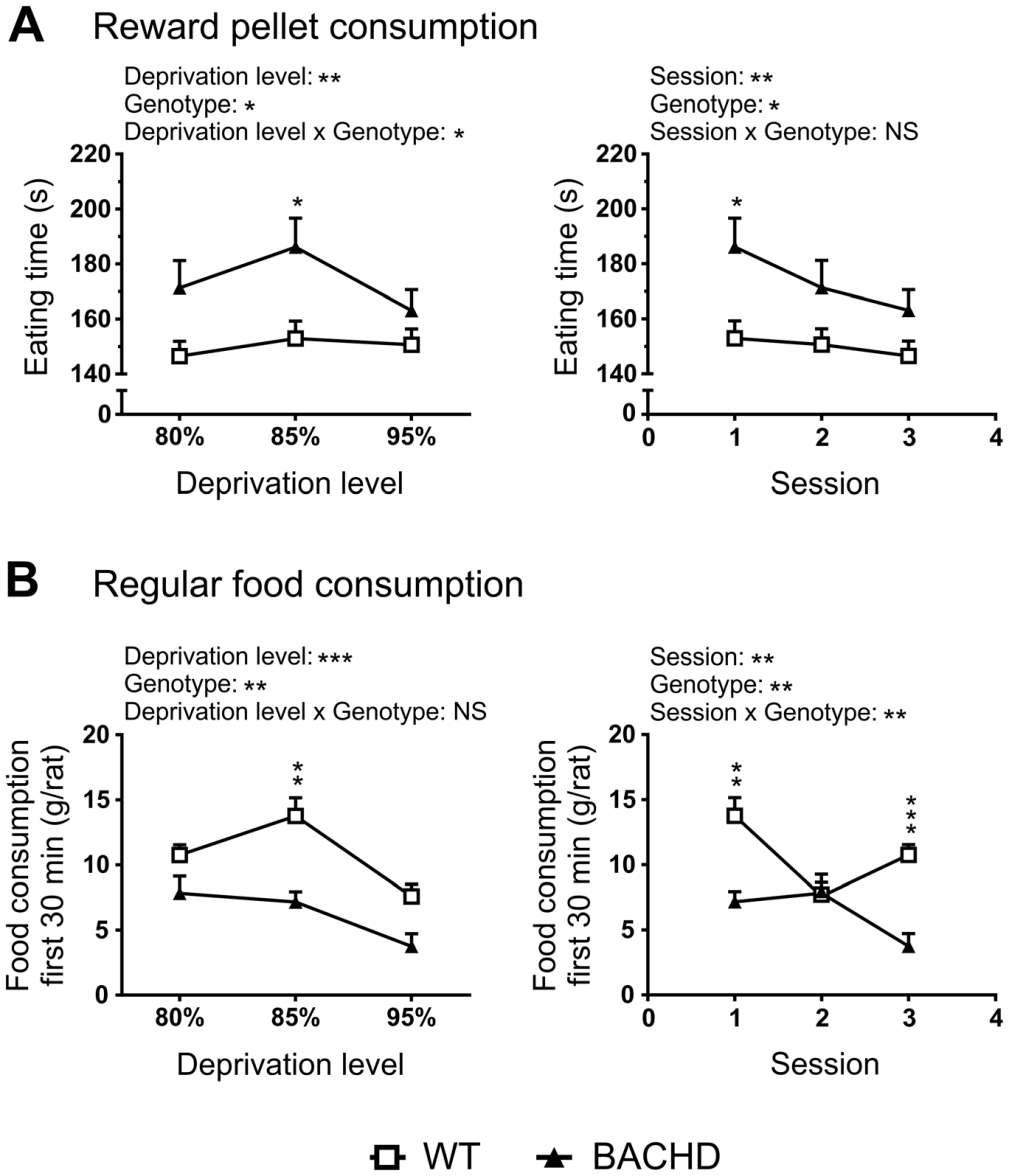
Impact of repeated testing and food deprivation on consumption tests. (**A**) The time needed to consume 100 reward pellets is plotted against the deprivation level (left graph) and session number (right graph). (**B**) The food consumed during the first 30 minutes of the regular food consumption test is plotted against the deprivation level (left graph) and session number (right graph). The graphs show mean plus standard error of the mean. Two-way ANOVA results are displayed above each graph, and results from *post-hoc* analysis are shown for individual data points. Significant genotype differences are indicated by (p<0.05) *, (p<0.01) **, (p<0.001) *** and (p<0.0001) ****.

A more detailed analysis of the results was performed with the aim of better assessing the impact of food deprivation levels on the consumption rate in the two tests. Separate two-way ANOVA analysis of the time needed to consume 100 reward pellets, using genotype as between-subject factor, and either food deprivation level or the number of test sessions as within-subject factor, revealed similar statistical results (Figure 5A). In either case, there was a significant genotype effect (p<0.05, F_(1,21)_  = 5.476), and performance on the first session, where both genotypes were deprived to 85%, differed significantly between genotype groups (p<0.05). Both analyses also revealed a significant effect of their respective within-subject parameter (p<0.01, F_(2,42)_  = 7.861 and 6.6333 for session and deprivation level, respectively). However, inspection of the graphed data indicated that the time needed to consume the reward pellets did not clearly decrease with increasing food deprivation levels, but did so with increased numbers of test sessions. Performing the same analyses on the amount of food consumed during the first 30 minutes of the food consumption test revealed different results (Figure 5B). Both analyses once again revealed a significant genotype effect (p<0.01, F_(1,6)_  = 15.59), and significant effects of their respective within-subject parameters (p<0.01, F_(2,12)_  = 8.220 and 17.04 for session and deprivation level, respectively). *Post-hoc* analysis of data analyzed in terms of food deprivation level revealed a significant difference in consumption rate when rats of both genotypes were deprived to 85% of their free-feeding body weight. This was also found when analyzing the data in terms of the number of test sessions given to the rats, although that analysis also revealed a significant difference in consumption rate during the third session. In contrast to the results from the pellet consumption test, the consumption rate in the food consumption test appeared to gradually increase with an increased food deprivation level, while not showing any gradual change during repeated testing.

### Progressive ratio performance during different levels of food deprivation

To better assess differences in the motivational state among the rats, a progressive ratio test was run with two different food deprivation settings. All rats learned to push the lever in order to obtain a reward pellet, although there were some discrete behavioral differences between WT and BACHD rats during the initial training steps. During habituation, BACHD rats made fewer entries into the pellet receptacle (Figure S3A, B) and were initially slower at retrieving the pellets (Figure S3C). During CRF, FR3 and FR5 training, BACHD rats were generally slower at both retrieving the pellets, and returning to the reinforced lever (Figure S4 and S5).

During the fixed ratio part of the PR protocol, BACHD rats were still slower at retrieving the reward pellets, but they no longer showed an increase in lever return latencies (Figure S6). These results were largely unaffected when food deprivation levels were adjusted. WT rats tended to take longer time to complete the FR5 ratios, although this became significant only after adjustment of their deprivation level (Figure S6). Importantly, there were no overt differences between genotypes in the overall response frequency on the rewarded lever during the fixed ratios (Figure S6). The same was true for the mean number of lever pushes made on the non-reinforced lever during the entire PR session (Figure S7).

**Figure 6:**
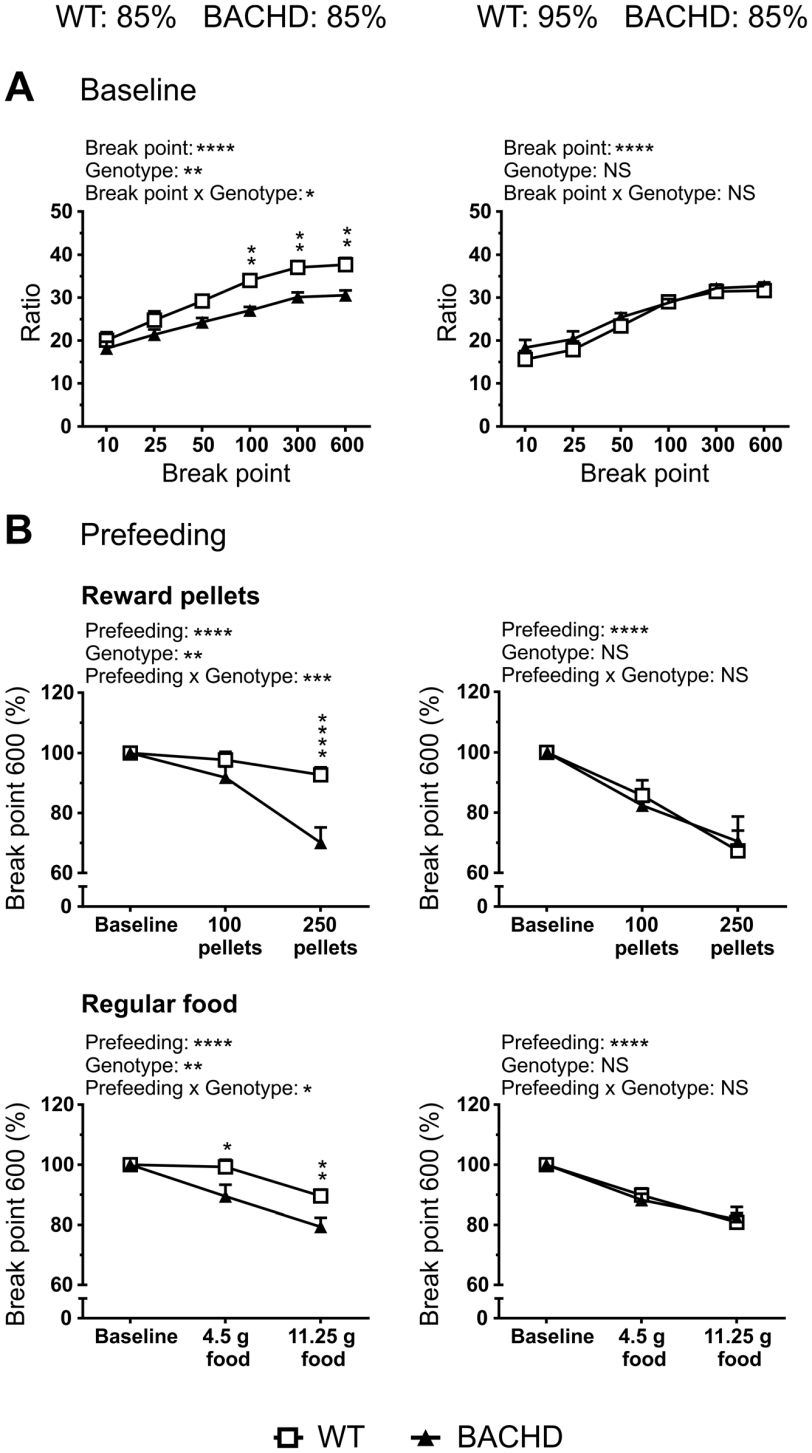
Progressive ratio test performance. Performance in the PR test is shown for when animals of both genotypes were deprived to 85% of their free-feeding body weight (graphs to the left in each figure panel) and when the deprivation level of WT rats had been adjusted to achieve equal food consumption rates between genotypes (graphs on the right of each figure panel). (**A**) Baseline performance during six consecutive PR sessions preceding the prefeeding tests. The ratio, where a given break point was reached, is indicated. (**B**) Performance during prefeeding with reward pellets (top panel) and regular food (bottom panel). The drop in motivation is displayed as percentage of baseline performance for break point 600. The graphs show group mean plus standard error of the mean. Two-way ANOVA results are displayed above each graph, and results from *post-hoc* analysis are shown for individual data points. Significant genotype differences are indicated by (p<0.05) *, (p<0.01) **, (p<0.001) *** and (p<0.0001) ****.

Analysis of how the rats reached a series of break points, when all were deprived to 85% of their free-feeding body weight, revealed both a significant genotype effect (p<0.01, F_(1,22)_  = 10.66) and differences in the three highest break points (p<0.01), with BACHD rats reaching lower ratios (Figure 6A). These differences were not present when the food deprivation level of WT rats had been adjusted so that their food consumption rate matched that of BACHD rats. Similarly, when all rats were deprived to 85% of their free-feeding body weight, BACHD rats responded with more pronounced drops in motivation during prefeeding of both reward pellets and regular food, as indicated by significant genotype effects (p<0.01, F_(1,22)_  = 9.461 and p<0.01, F_(1,21)_  = 8.343 for reward pellet and regular food prefeeding, respectively) and prefeeding x genotype interactions (p<0.001, F_(2,44)_  = 11.19 and p<0.05, F_(1,21)_  = 8.341 for reward pellet and regular food prefeeding, respectively) (Figure 6B). Once again, these phenotypes were not present when the food deprivation level of WT rats had been adjusted, leading to identical responses in the prefeeding tests. It should be noted that only the last break point, break point 600, was suitable for prefeeding analysis. Prefeeding induced a strong interest in water among WT rats, which dramatically affected their early break points (data not shown). It should also be noted that there was a significant difference in body weight once the food deprivation levels had been adjusted, with WT rats being significantly heavier than BACHD rats (data not shown). The WT rats weighed roughly 50 g more than BACHD rats, resulting in them being at 95% of their free-feeding body weight.

## Discussion

### Body composition and food intake of BACHD rats

Many transgenic animal models of HD show an altered body weight compared to their WT littermates. Animals that express a fragment of the disease-causing gene typically have a reduced body weight [25], [26], [27], while the ones that express the full-length gene typically have an increased body weight [10], [11]. We show here, that although BACHD rats did not differ from WT rats in terms of body weight, they displayed several changes in body composition. Strikingly, BACHD rats carried an excess amount of adipose tissue. This is in line with phenotypes of other full-length models of HD, as the increased body weight of BACHD and YAC128 mice has been shown to at least in part be due to an increase in adipose tissue mass [28], [29]. It should be pointed out that R6/2 and N171-82Q mice, which only express a fragment of the disease-causing gene, also carry excess amounts of adipose tissue [25], [30]. R6/2 mice have further been shown to maintain this increased fat mass even when they start to lose weight [25]. Thus, the increase in adipose tissue seems to be a common phenotype of transgenic HD models, although it does not always result in obesity.

Increased amounts of adipose tissue could theoretically be the result of increased food intake, decreased home cage activity, metabolic disturbances, or a combination of the three. While BACHD mice have been shown to eat more than their WT littermates [28], R6/2 and YAC128 mice have been found to have unchanged food intake [25], [29]. A previous study on BACHD rats, in which food intake was followed from three to eighteen months of age, indicated that the transgenic rats ate less than their WT littermates [22]. These results were well reproduced here, despite the different housing conditions. The current study also assessed food intake at ages younger than three months, where BACHD rats appeared to consume more food compared to WT rats. It should be noted, however, that the appearance of the food consumption phenotypes was to some degree dependent on whether or not the weight of the consumed food was normalized to the animals' body weight. The aim of this normalization was to relate the rats' food intake to a measurement of their body size, and through this investigate if the reduced food intake among BACHD rats could be due to them being smaller than WT rats. Using body weight as an approximation of body size is, however, probably only suitable at young ages, as the body weight of older BACHD rats is distorted due to obesity. Thus, further studies are needed to reach conclusions on this matter. In addition, as food intake phenotypes are unlikely to explain the increase in adipose tissue, metabolic parameters of BACHD rats need to be further characterized. In this regard, it is important to note that the obesity phenotype of BACHD mice was abolished when the expression of mutant Huntingtin was silenced in the hypothalamus [28]. Interestingly, hypothalamic lesions can induce obesity that is not always associated with increased food intake, but can persist despite unchanged or even reduced food intake [31], [32], [33], [34], [35]. The differential effects appear to depend on which specific neuronal population is damaged [35], [36], which might relate to the common phenotype of increased fat mass, but varied food intake seen across HD animal models.

In the current study, BACHD rats were shown to have a smaller body size and disproportionately lower amount of bone/muscle tissue compared to WT rats. Information about similar parameters is scarce for other HD models, although YAC128 mice have been shown to have unchanged lean body mass [29], while R6/2 mice show a progressive reduction in lean body mass as they age [25]. These are both in contrast to the bone/muscle phenotype seen in BACHD rats, as the lower amount of bone/muscle tissue seen in the current study did not seem to progress with age. Instead, the body size and bone/muscle phenotypes seen in the BACHD rats appeared to be caused by discrete developmental deficits and stunted growth. It is unlikely that these phenotypes were the result of malnutrition during testing, as food was available *ad libitum* on the cage floor. It is possible, however, that BACHD pups might have had difficulties when competing for mothers' milk, leading to malnutrition at early ages. Such factors have been shown to affect the growth of animals from large litters [37]. Alternatively, the growth of BACHD rats might be disturbed on a molecular level, as Huntingtin has been shown to be important during fetal development [38]. The fact that BACHD rats had smaller heads compared to WT rats is particularly interesting, as similar symptoms have been seen in HD gene-carriers [39]. Thus, the discrete developmental deficits found in the BACHD rats might be closely connected to developmental deficits of human patients.

### Food deprivation and motivation of BACHD rats

Behavioral assessment of HD animal models through the use of operant conditioning tests is of interest, as cognitive symptoms are common in HD patients and might become valuable to clinically track disease progression and treatment effects [40], [41], [42]. Many conditioning protocols require food deprivation in order to both efficiently train the animals to perform a given task and to maintain high performance. However, food deprivation of HD models requires extra care as they can be expected to have changes in body composition. To better understand how to optimally food deprive BACHD rats, we assessed their interest in food in a total of three different tests.

Free intake of reward pellets and regular food is sometimes used to assess an animal's hunger level and interest in food [18], [19], [20], [21]. In the current study, WT and BACHD rats deprived to 85% of their free-feeding body weight did not seem to differ in their interest in consuming 100 reward pellets, although BACHD rats needed more time to eat all pellets. Food deprivation levels were then adjusted in an attempt to reverse the phenotypes, however, this did not seem to affect the rats' behavior. Instead, both the time spent exploring the arena and the time needed to consume all pellets decreased with repeated testing. The training effect on the consumption rate eventually led to BACHD rats consuming the reward pellets at an equal rate compared to WT rats. There were indications that rats deprived to 95% of their free-feeding body weight spent more time exploring the arena compared to rats deprived to 80%, but this generally concerned one or two rats of an entire group of twelve. As the current protocol did not appear to be sensitive even to large changes in food deprivation levels, it is unlikely to be a suitable test for assessing discrete differences in food interest. It is also clear that the apparent training effect could be misinterpreted as a food deprivation effect, if one assessed a given group of animals repeatedly with the aim of gradually adjusting their food deprivation level. The slowed consumption speed seen among BACHD rats in the pellet consumption test is, however, an interesting phenotype on its own. While eating, rats typically stood on all four paws and used their tongue to pick up the pellets. Thus, the slower feeding rate among BACHD rats is likely due to impairments in quite basic processes that are needed for eating. These could include impaired chewing, swallowing or tongue movements as well as reduced saliva production. It is tempting to hypothesize that the slower feeding speed among BACHD rats could be due to phenotypes similar to the tongue protrusion symptoms that are often seen among HD patients [43], [44]. Interestingly, there are protocols for measuring tongue protrusion [45] in rats, although these tests must be performed carefully, as the smaller head size of BACHD rats likely means that they have shorter tongues as well.

In the regular food consumption test, BACHD rats consumed less food than WT rats when both groups were deprived to 85% of their respective free-feeding body weight. Consumption rate during the first 30 minutes of the test changed in a predictable way when deprivation levels were adjusted, with more deprived rats eating at a faster rate. This suggests that the protocol was well suited for the assessment of food interest and hunger levels. Our results further showed that when BACHD and WT rats were deprived to 80 and 95% of their respective free-feeding body weights, they consumed food at an identical rate for the initial 150 minutes, indicating that the rats were equally hungry. As the test session continued, BACHD rats once again ate less than WT rats, which likely reflected differences in the rats' satiety levels. It should be noted that the feeding behavior of either genotype did not significantly differ when comparing their 80 and 85% food deprivation test sessions. Thus, although the test seems suitable to assess food interest, it does not appear to be very sensitive. Assessing food consumption in single animals, rather than in groups, would most likely improve the test's sensitivity. It would further allow separate scoring of the time spent eating and the time spent not eating, as it was done in the reward pellet consumption test. However, despite extensive habituation, we have found it difficult to get our rats to efficiently consume regular food in any other setup than their home cages. As the test did not allow separate scoring of the time the rats spent feeding and doing other activities, it was not possible to conclude if the difference in consumption rate was strictly due to a difference in hunger and food interest. This idea is especially difficult to support when considering the results of the pellet consumption test. In an attempt to reach a conclusion on the matter, we ran a PR test with prefeedings.

When both WT and BACHD rats were deprived to 85% of their respective free-feeding body weight, BACHD rats were clearly less motivated to work for food rewards in the PR test. Similar phenotypes have been found in other HD models [16], [46] and they are typically discussed in terms of apathy, which is a common symptom among HD patients [47], [48]. However, BACHD rats also responded with more pronounced drops in motivation during the prefeeding tests, which would typically be interpreted as BACHD rats being less hungry compared to WT rats [49], [50], [51]. This would also support the idea that the BACHD rats' lower consumption rate in the first session of the food consumption test was to some degree caused by lower hunger and food interest. When the food deprivation level of WT rats was adjusted to achieve equal food consumption rates to those of the BACHD rats, all genotype differences that were previously seen in the PR test disappeared. As WT and BACHD rats did not differ during prefeeding tests, it is reasonable to assume that they were equally hungry and that the food consumption test was suitable for establishing food deprivation levels that ensured this. As they also no longer differed in baseline performance, the motivational deficit seen in the first PR test was likely dependent on a difference in hunger levels, rather than an apathy-related phenotype. It is interesting to note that after the food deprivation levels had been adjusted, BACHD rats weighed approximately 50 g less than WT rats. This difference was similar to the one found in bone/muscle tissue, suggesting that WT and BACHD rats carried a similar amount of adipose tissue. Secretion of leptin, which affects satiety and food intake [52], [53], is proportional to adipose tissue mass [54], and it is possible that the food deprivation adjustment led to equal hunger and food interest due to equal levels of leptin. Importantly, higher leptin levels have been shown to reduce motivation in PR tests [55], which gives a possible explanation for the initial motivational difference.

Most of the conclusions above are based on the idea that prefeeding responses depend exclusively on hunger levels and not on other aspects of motivation. One could argue that animals that suffer from motivational deficits not related to hunger, might also respond stronger on the prefeeding tests. Thus, seeking a situation where animals respond equally to prefeeding could in itself lead to the lack of differences in PR performance. It is therefore important to note that other studies have found motivational differences despite identical responses on prefeeding tests [51], and that motivational deficits have been found in BACHD mice after adjusting deprivation levels until animals consumed food at the same rate [16]. It should also be noted that the true nature of the motivational phenotype seen here is mainly of importance when such phenotypes are being characterized. If one simply wishes to minimize motivational differences when working with BACHD rats, regardless if these are due to hunger levels or other aspects of motivation, adjusting deprivation levels so that WT and BACHD rats consume regular food at a comparable rate should suffice. Still, the current study only considered quite young animals. It is possible that older BACHD rats suffer from motor impairments that could affect the validity of the food consumption test. Also, motivational phenotypes not related to hunger might become apparent among older BACHD rats. We aim at addressing these ideas in a longitudinal study of PR performance.

### Summary

In the current study, BACHD rats were found to have metabolic disturbances, which is in line with other animal models of HD. We further found that unless these phenotypes were taken into consideration during food deprivation, BACHD rats were less motivated than WT rats in a progressive ratio test. Thus, metabolic phenotypes are important to consider as possible confounding factors when assessing apathy-related phenotypes of BACHD rats. The same is likely true for other HD animal models with metabolic abnormalities.

Our results further indicated that basing the animals' food deprivation levels on their consumption rates of regular food was a convenient way to avoid motivational differences between BACHD and WT rats. Thus, previous studies that applied this method when studying apathy in HD animal models [16] likely avoided hunger-based motivational differences, and our results support the future use of this method. It is also important to consider its use in behavioral tests where the main readout is not directly related to apathy or motivation, such as [17], as motivational differences have been shown to affect animals' behavior in such tests too [15].

## Supporting Information

Figure S1 **Food debris and water consumption during the** ***ad libitum*** **food consumption test.** (**A**) The approximate daily amount of food debris produced per cage (calculated from a three- to four-day average), plotted against the age of the rats. (**B**) The approximate amount of food debris per cage relative to the average food consumption per cage, plotted against the age of the rats. (**C**) The approximate daily food consumption per rat (calculated from the weekly food consumption per cage) after accounting for food debris left in the cages, plotted against the age of the rats. (**D**) The approximate daily water consumption per rat (calculated from the weekly water consumption per cage), plotted against the age of the rats. The graphs indicate group mean plus standard error of the mean. Two-way ANOVA results are displayed above each graph, and results from *post-hoc* analysis are shown for individual data points. Significant genotype differences are indicated by (p<0.05) *, (p<0.01) **, (p<0.001) *** and (p<0.0001) ****. For (**D**), WT and BACHD rats differed highly significant (****) for all data points between 11 and 26 weeks of age.
(TIF)

Figure S2 **Body length measurements.** (**A–D**) Data from length measurement as stated in the graph titles. The graphs show group mean plus standard error of the mean. Two-way ANOVA results are displayed above each graph, and significant results from *post-hoc* analysis are displayed inside each graph. Significant genotype differences are indicated by (p<0.05) *, (p<0.01) **, (p<0.001) *** and (p<0.0001) ****.
(TIF)

Figure S3 **Habituation to the operant conditioning boxes.** (**A**) The total number of head entries made into the pellet receptacle during habituation sessions. (**B**) The total time spent with the head inside of the pellet receptacle during habituation sessions as a measurement of the duration of receptacle visits. (**C**) The mean latency to enter the pellet receptacle after the delivery of a reward pellet. The graphs indicate group mean plus standard error of the mean. Two-way ANOVA results are displayed above each graph, and results from *post-hoc* analysis are shown for individual data points. Significant genotype differences are indicated by (p<0.05) *, (p<0.01) **, (p<0.001) *** and (p<0.0001) ****.
(TIF)

Figure S4 **Performance on the CRF protocol.** Results from the final session of CRF training are shown as indicated by graph titles. Session duration measured the time the rats needed to complete 100 ratios. Retrieval latency measured the time between the release of the reinforced lever and the entry into the pellet receptacle. Lever return latency was defined as the interval between the first receptacle entry following reward delivery and the lever push that followed. Graphs indicate the performance of individual rats and group mean. Results from t-tests or Mann-Whitney tests are indicated in the graphs. Significant genotype differences are indicated by (p<0.05) *, (p<0.01) **, (p<0.001) *** and (p<0.0001) ****.
(TIF)

Figure S5 **Performance on fixed ratio protocols.** Results for several basic parameters of FR3 and FR5 protocols are shown as indicated by the graph titles. Session duration measured the time the rats needed to complete 100 ratios. Ratio duration measured the time between the first and last lever push of each ratio. Ratio interval was defined as the time between the last lever push of one ratio and the first lever push of the ratio that followed. Retrieval latency measured the time between the release of the reinforced lever and the entry into the pellet receptacle. Lever return was defined as the interval between the first receptacle entry following reward delivery and the first lever push of the ratio that followed. Scatter plots of FR3 results indicate the performance of individual rats and group mean. Results from t-tests or Mann-Whitney tests are indicated in the graphs. Only results from the final session, where rats performed at criterion, are displayed. Line graphs of FR5 results indicate group mean plus standard error of the mean, plotted against the training session. Only the three final sessions, where rats performed at criterion, are included. Two-way ANOVA results are displayed at the top right corner of each FR5 graph, and significant results from *post-hoc* analysis are shown for individual data points. Significant genotype differences are indicated by (p<0.05) *, (p<0.01) **, (p<0.001) *** and (p<0.0001) ****.
(TIF)

Figure S6 **Performance on the fixed ratio part of the progressive ratio protocol.** Results for the basic parameters of the ten FR5 ratios run at the start of each PR session. (**A**) Data from sessions where BACHD and WT rats were both deprived to 85% of their respective free-feeding body weights. (**B**) Data from sessions where food deprivation was adjusted to match the food consumption rate of BACHD and WT rats. Details for each parameter are described in the figure legend of Figure S4 and S5. Lever push frequency was calculated based on the pushes made on the reinforced lever during the full length of a ratio, i.e. the ratio duration plus interval to subsequent ratio. Results displayed were obtained from the sessions used for baseline curves in Figure 6A. The graphs indicate group mean plus standard error of the mean. Two-way ANOVA results are displayed at the top right corner of each graph, and results from *post-hoc* analysis are shown for individual data points. Significant genotype differences are indicated by (p<0.05) *, (p<0.01) **, (p<0.001) *** and (p<0.0001) ****.
(TIF)

Figure S7 **Mean number of errors for the fixed ratio part of the progressive ratio protocol.** Errors made by the rats during the ten FR5 ratios run at the start of each PR session. (**A**) Data from sessions where BACHD and WT rats were both deprived to 85% of their respective free-feeding body weights. (**B**) Data from sessions where food deprivation was adjusted to match the food consumption rate of BACHD and WT rats. Results were obtained from the sessions used for baseline curves in Figure 6A. Graphs indicate the performance of individual rats and group mean. Results from t-tests or Mann-Whitney tests are indicated in the graphs. Significant genotype differences are indicated by (p<0.05) *, (p<0.01) **, (p<0.001) *** and (p<0.0001) ****.
(TIF)
