# Supplementary figures and images for: Reduced Motivation in the BACHD Rat Model of Huntington Disease Is Dependent on the Choice of Food Deprivation Strategy
Source: PLoS One. 2014 Aug 21;9(8):e105662. doi: 10.1371/journal.pone.0105662 (PMC4140820; doi:10.1371/journal.pone.0105662)

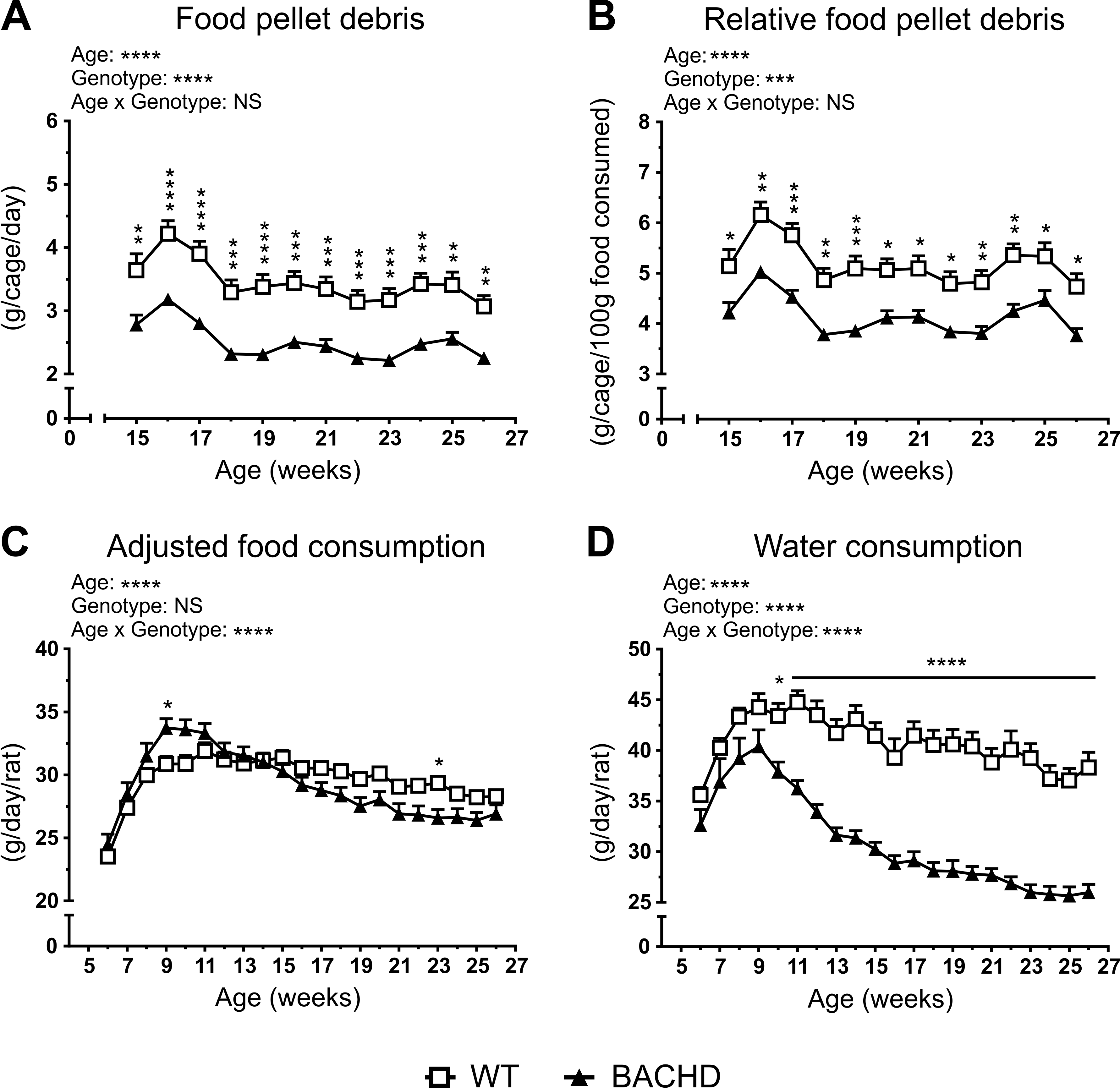

Supplement: Figure S1 — Food debris and water consumption during the ad libitum food consumption test. (A) The approximate daily amount of food debris produced per cage (calculated from a three- to four-day average), plotted against the age of the rats. (B) The approximate amount of food debris per cage relative to the average food consumption per cage, plotted against the age of the rats. (C) The approximate daily food consumption per rat (calculated from the weekly food consumption per cage) after accounting for food debris left in the cages, plotted against the age of the rats. (D) The approximate daily water consumption per rat (calculated from the weekly water consumption per cage), plotted against the age of the rats. The graphs indicate group mean plus standard error of the mean. Two-way ANOVA results are displayed above each graph, and results from post-hoc analysis are shown for individual data points. Significant genotype differences are indicated by (p<0.05) *, (p<0.01) **, (p<0.001) *** and (p<0.0001) ****. For (D), WT and BACHD rats differed highly significant (****) for all data points between 11 and 26 weeks of age. (TIF) [file pone.0105662.s001.tif]

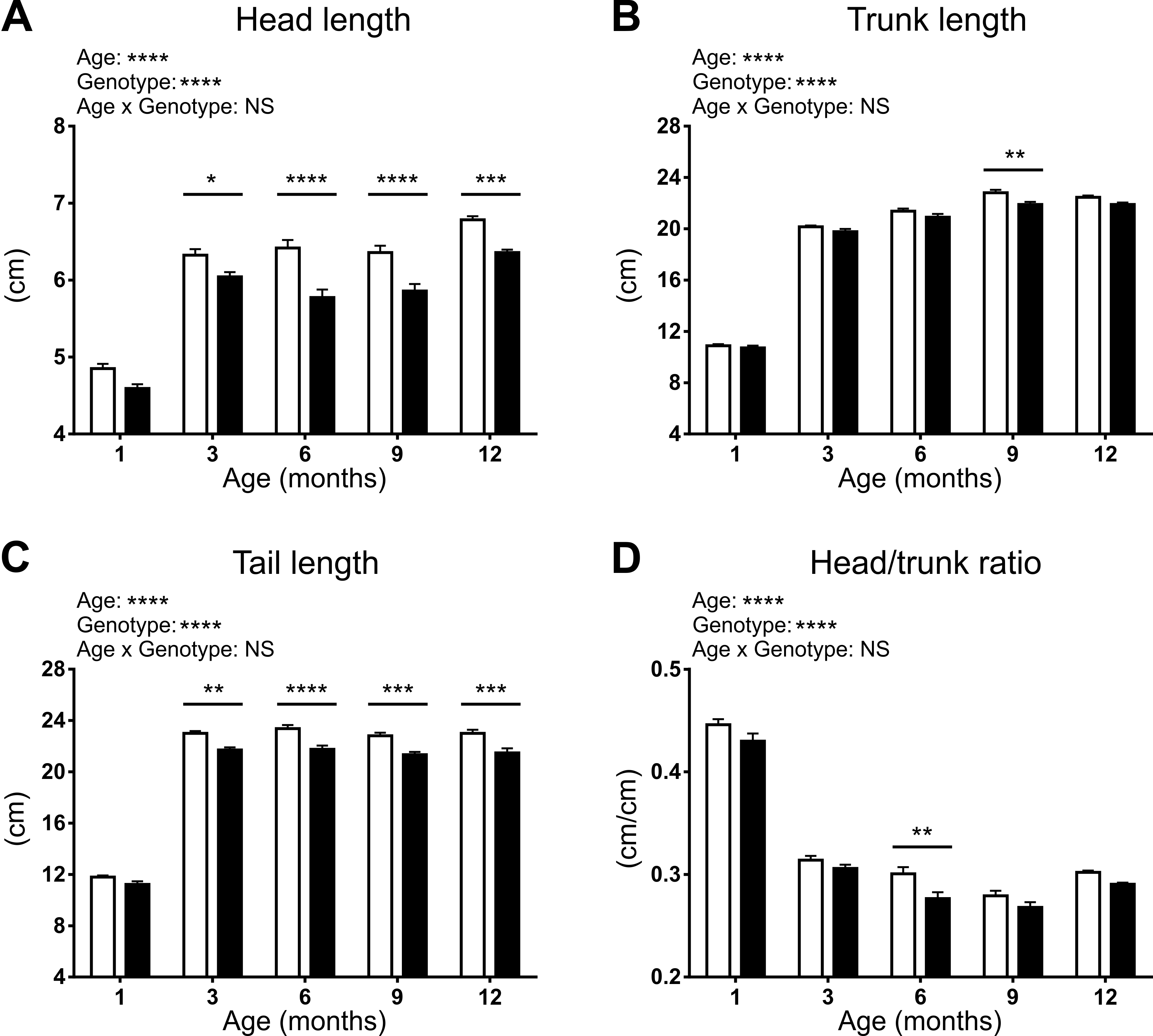

Supplement: Figure S2 — Body length measurements. (A–D) Data from length measurement as stated in the graph titles. The graphs show group mean plus standard error of the mean. Two-way ANOVA results are displayed above each graph, and significant results from post-hoc analysis are displayed inside each graph. Significant genotype differences are indicated by (p<0.05) *, (p<0.01) **, (p<0.001) *** and (p<0.0001) ****. (TIF) [file pone.0105662.s002.tif]

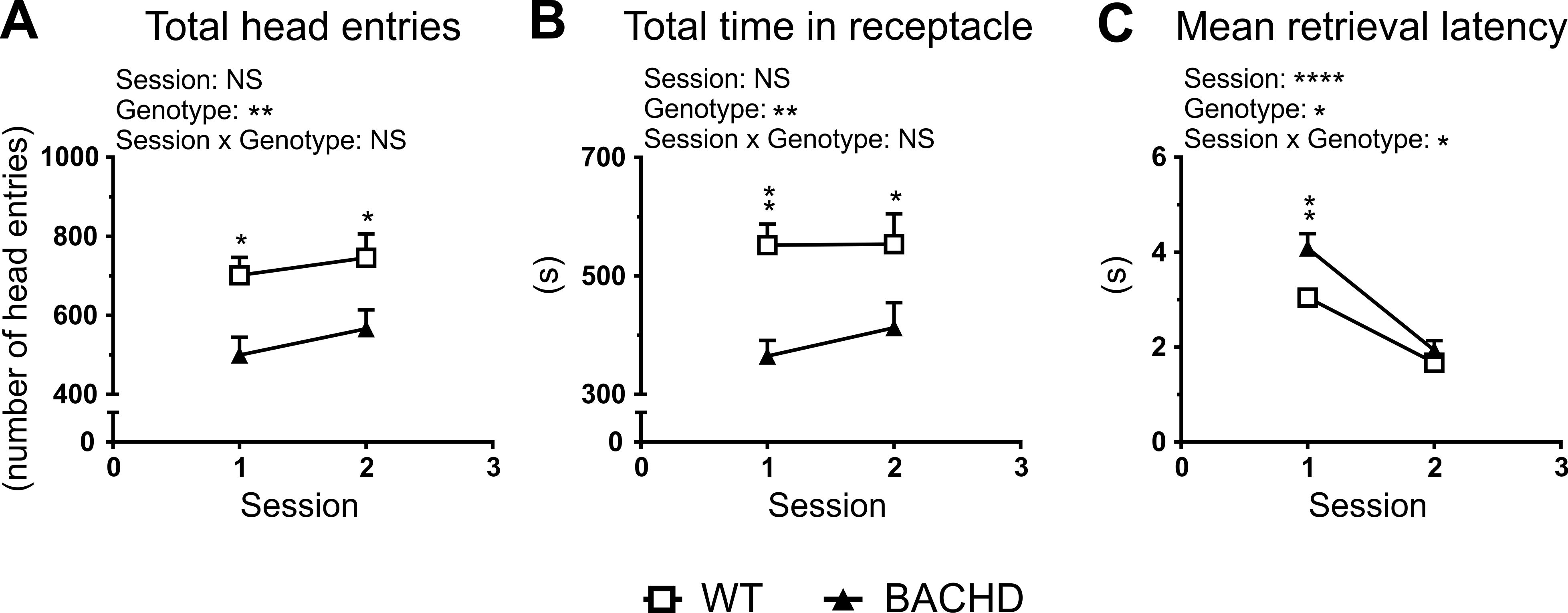

Supplement: Figure S3 — Habituation to the operant conditioning boxes. (A) The total number of head entries made into the pellet receptacle during habituation sessions. (B) The total time spent with the head inside of the pellet receptacle during habituation sessions as a measurement of the duration of receptacle visits. (C) The mean latency to enter the pellet receptacle after the delivery of a reward pellet. The graphs indicate group mean plus standard error of the mean. Two-way ANOVA results are displayed above each graph, and results from post-hoc analysis are shown for individual data points. Significant genotype differences are indicated by (p<0.05) *, (p<0.01) **, (p<0.001) *** and (p<0.0001) ****. (TIF) [file pone.0105662.s003.tif]

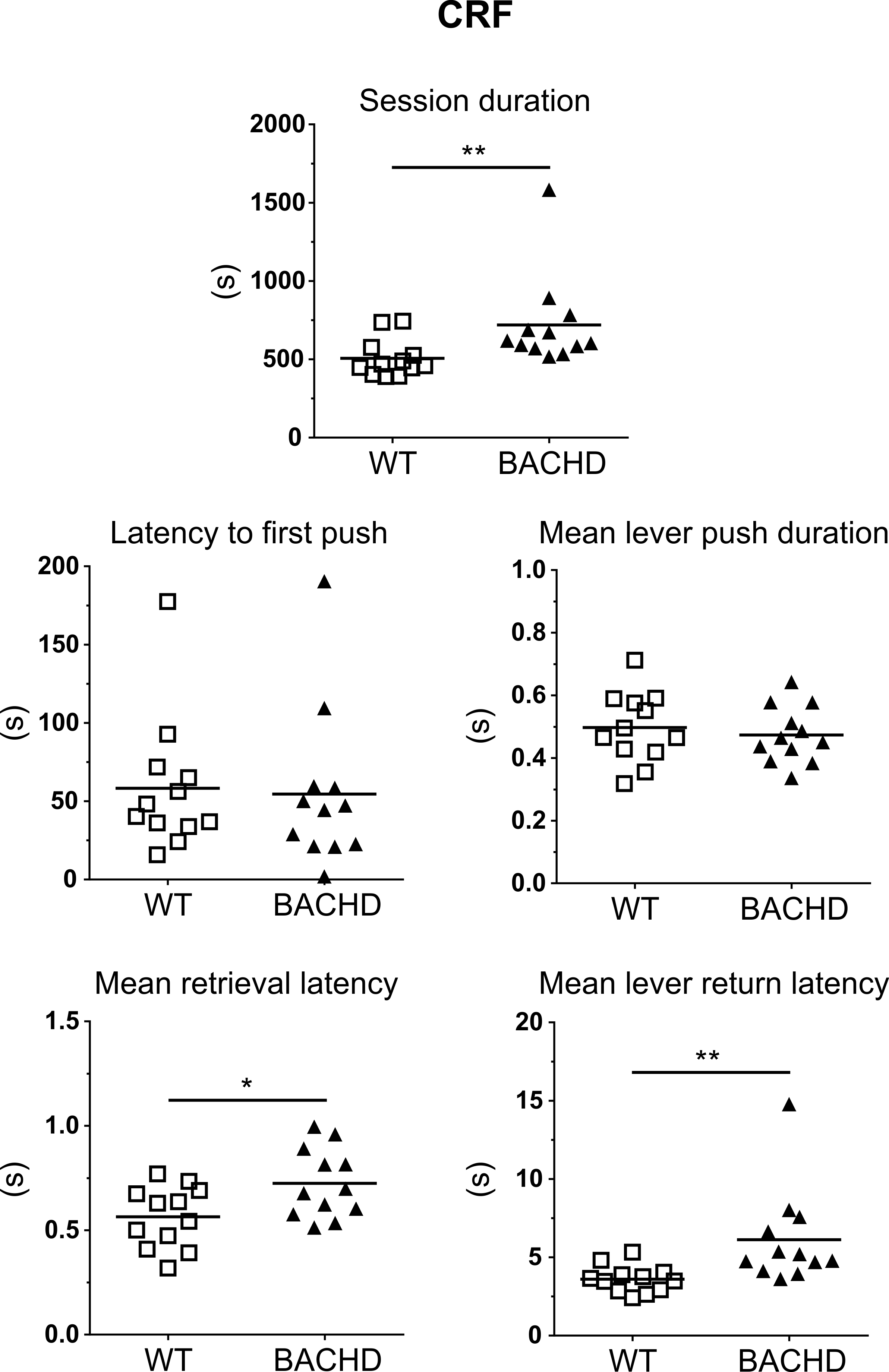

Supplement: Figure S4 — Performance on the CRF protocol. Results from the final session of CRF training are shown as indicated by graph titles. Session duration measured the time the rats needed to complete 100 ratios. Retrieval latency measured the time between the release of the reinforced lever and the entry into the pellet receptacle. Lever return latency was defined as the interval between the first receptacle entry following reward delivery and the lever push that followed. Graphs indicate the performance of individual rats and group mean. Results from t-tests or Mann-Whitney tests are indicated in the graphs. Significant genotype differences are indicated by (p<0.05) *, (p<0.01) **, (p<0.001) *** and (p<0.0001) ****. (TIF) [file pone.0105662.s004.tif]

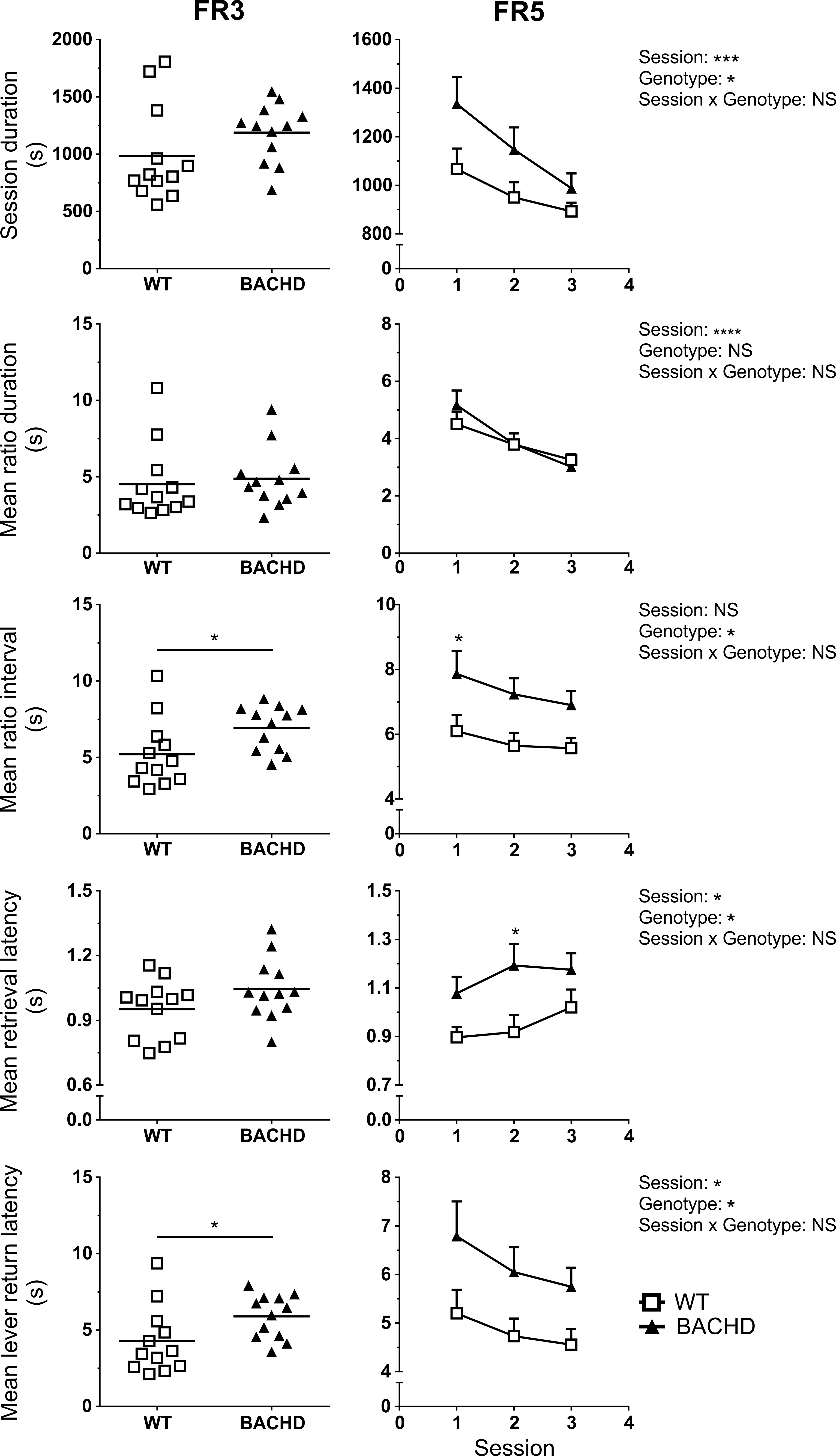

Supplement: Figure S5 — Performance on fixed ratio protocols. Results for several basic parameters of FR3 and FR5 protocols are shown as indicated by the graph titles. Session duration measured the time the rats needed to complete 100 ratios. Ratio duration measured the time between the first and last lever push of each ratio. Ratio interval was defined as the time between the last lever push of one ratio and the first lever push of the ratio that followed. Retrieval latency measured the time between the release of the reinforced lever and the entry into the pellet receptacle. Lever return was defined as the interval between the first receptacle entry following reward delivery and the first lever push of the ratio that followed. Scatter plots of FR3 results indicate the performance of individual rats and group mean. Results from t-tests or Mann-Whitney tests are indicated in the graphs. Only results from the final session, where rats performed at criterion, are displayed. Line graphs of FR5 results indicate group mean plus standard error of the mean, plotted against the training session. Only the three final sessions, where rats performed at criterion, are included. Two-way ANOVA results are displayed at the top right corner of each FR5 graph, and significant results from post-hoc analysis are shown for individual data points. Significant genotype differences are indicated by (p<0.05) *, (p<0.01) **, (p<0.001) *** and (p<0.0001) ****. (TIF) [file pone.0105662.s005.tif]

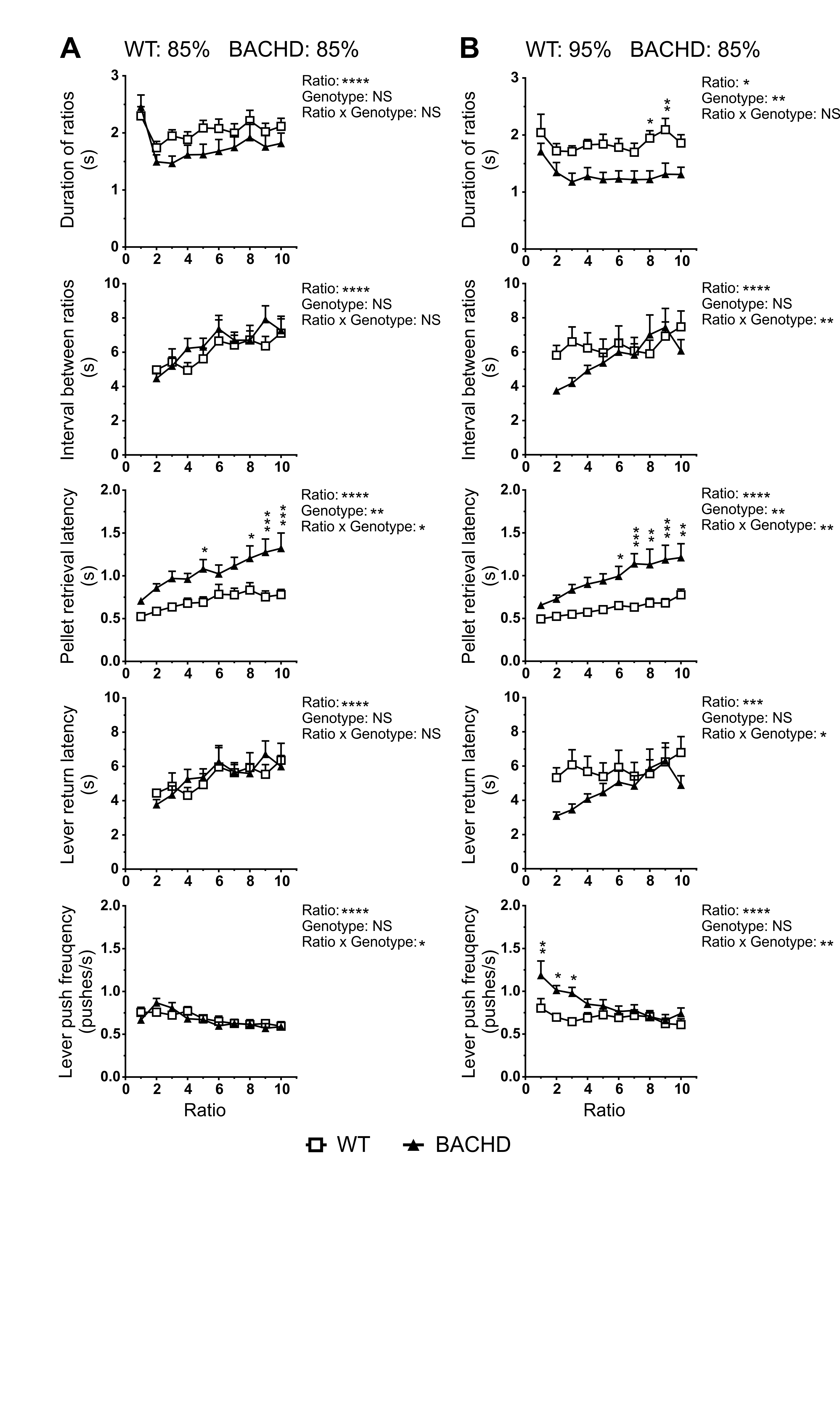

Supplement: Figure S6 — Performance on the fixed ratio part of the progressive ratio protocol. Results for the basic parameters of the ten FR5 ratios run at the start of each PR session. (A) Data from sessions where BACHD and WT rats were both deprived to 85% of their respective free-feeding body weights. (B) Data from sessions where food deprivation was adjusted to match the food consumption rate of BACHD and WT rats. Details for each parameter are described in the figure legend of Figure S4 and S5. Lever push frequency was calculated based on the pushes made on the reinforced lever during the full length of a ratio, i.e. the ratio duration plus interval to subsequent ratio. Results displayed were obtained from the sessions used for baseline curves in Figure 6A. The graphs indicate group mean plus standard error of the mean. Two-way ANOVA results are displayed at the top right corner of each graph, and results from post-hoc analysis are shown for individual data points. Significant genotype differences are indicated by (p<0.05) *, (p<0.01) **, (p<0.001) *** and (p<0.0001) ****. (TIF) [file pone.0105662.s006.tif]

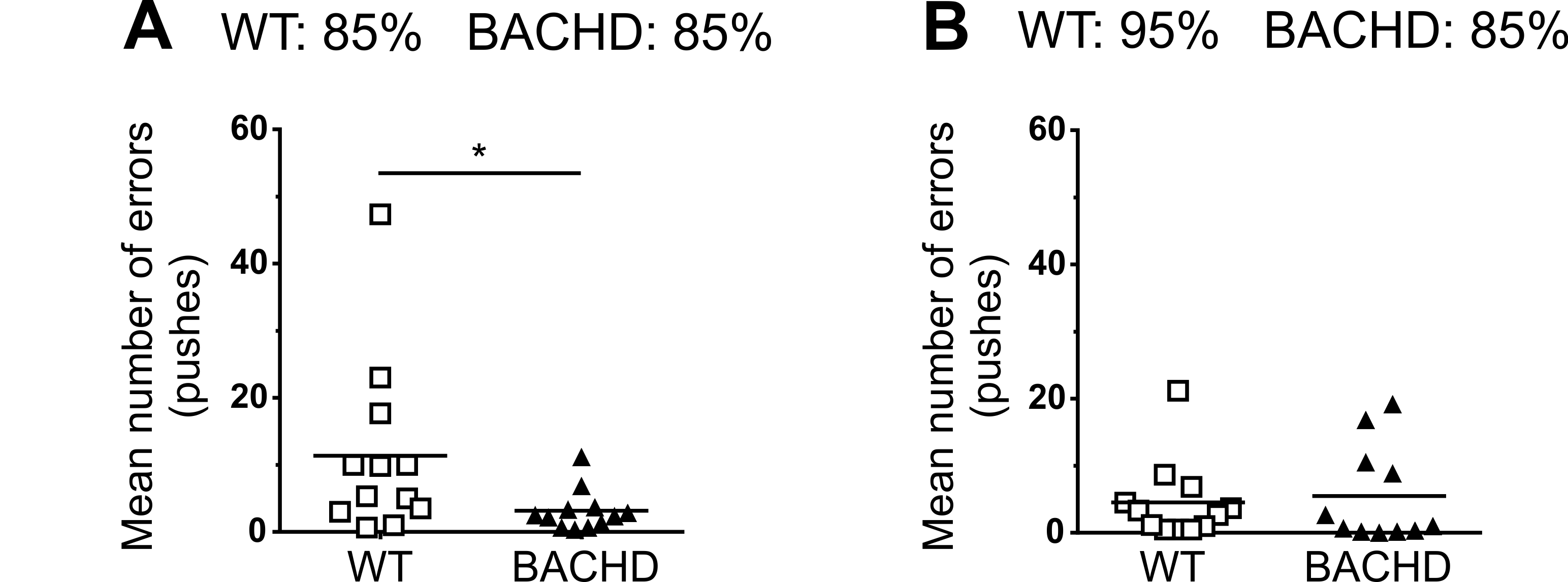

Supplement: Figure S7 — Mean number of errors for the fixed ratio part of the progressive ratio protocol. Errors made by the rats during the ten FR5 ratios run at the start of each PR session. (A) Data from sessions where BACHD and WT rats were both deprived to 85% of their respective free-feeding body weights. (B) Data from sessions where food deprivation was adjusted to match the food consumption rate of BACHD and WT rats. Results were obtained from the sessions used for baseline curves in Figure 6A. Graphs indicate the performance of individual rats and group mean. Results from t-tests or Mann-Whitney tests are indicated in the graphs. Significant genotype differences are indicated by (p<0.05) *, (p<0.01) **, (p<0.001) *** and (p<0.0001) ****. (TIF) [file pone.0105662.s007.tif]
